# First-Principle Calculation of High Absorption-TlGaTe_2_ for Photovoltaic Application

**DOI:** 10.3390/ma12172667

**Published:** 2019-08-22

**Authors:** Murugesan Rasukkannu, Dhayalan Velauthapillai, Ponniah Vajeeston

**Affiliations:** 1 Department of Computing, Mathematics and Physics, Western Norway University of Applied Sciences, Inndalsveien 28, Box 5063, 5003-5268 Bergen, Norway; 2 Center for Materials Science and Nanotechnology, Department of Chemistry, University of Oslo, Box 1033 Blindern N-0315, 0001-1299 Oslo, Norway

**Keywords:** HSE06, PV materials, hybrid functional, TlGaTe_2_, absorption material

## Abstract

We use first-principle calculations based on hybrid functional and the Bethe-Salpeter equation method to investigate the electronic and optical properties of dichalcogenide TlGaTe_2_. Based on theoretical studies, TlGaTe_2_ has until recently been considered as an indirect band gap material, however; by employing more accurate hybrid functional model, we showed that although TlGaTe_2_ has an indirect band gap of 1.109 eV, it also exhibits a fundamental direct band gap of 1.129 eV. Our finding was further confirmed by the optical studies on TlGaTe_2_, which show that the absorption peak is registered at a photon energy of 1.129 eV. It was also shown that TlGaTe_2_ has high optical absorption peaks in the visible region. Based on phonon and elastic constant calculations, it was shown that TlGaTe_2_ is dynamically and mechanically stable. Our findings show that TlGaTe_2_ is a potential candidate for photovoltaic application.

## 1. Introduction

Photovoltaics (PV) show great potential in meeting the needs of the world’s energy consumption. Silicon-based solar cell technologies have over 90% of the PV market today, but the research community has been yearning for new materials that are cheap, flexible and efficient in solar cells. Band gap value and absorption coefficient of the material are the two major factors that decide whether the material can be of interest for photovoltaic (PV) applications. We have earlier reported several promising non-silicon materials with intermediate band gaps for photovoltaic applications [1,2]. There is an increasing interest in the family of ternary compounds with the general formula III-III-VI_2_ (where III = Al, Ga, In, Tl; VI = Se, Te, S)—known as dichalcogenides [3]. In a recent published article, we carried out in-depth analysis of TlBiS_2_ (thallium-based) and calculated electronic and optical properties for the photovoltaic applications [4]. Thallium-bearing ternary materials have been studied in optoelectronics [5], but not extensively investigated in photovoltaics due to the fact that thallium is toxic. Researchers have shown interest to the Hall effect analysis, the photoconductivity and recombination kinetics in dichalcogenide-TlGaTe_2_ material [6,7]. From our earlier study on non-silicon solar cell materials [8], we identified that TlGaTe_2_ has a strong absorption coefficient in the visible region, but a complete study of TlGaTe_2_ is needed to confirm that it is potential material for PV applications. 

In this article, we study structural, electronic, spectroscopic and optical properties of TlGaTe_2_ dichalcogenide using first-principle calculations. As the density functional theory (DFT) is considered as one of the most effective and accurate computational methods, we investigate the electronic band structure of TlGaTe_2_ employing DFT analysis. We report accurate electronic band structure of TlGaTe_2_ based on a hybrid density functional (HSE06) calculations. The calculated HSE06 band structure for this compound helps to identify the type of band gap TlGaTe_2_ possess and its value, authenticating the suitability for the solar cell application. We also study the structural stability and mechanical stability of TlGaTe_2_. To verify the Raman active (R-active) and infrared active (IR-active) modes with previously published experimental results [9], we simulated the Raman spectra and infrared spectra of TlGaTe_2_.

## 2. Computational Details

Total energies of TlGaTe_2_ were determined by the projected augmented plane-wave (PAW) utilization of the Vienna ab initio simulation package (VASP) [10]. We used the Perde-Burke-Ernzerhof (PBE) of the generalized gradient approximation (GGA) to achieve the structure relaxations [11]. The ionic coordinates are fully relaxed until the total energy is changed 10^−6^ eV per atom. Ground-state geometries were calculated by minimizing stresses and Hellman-Feynman forces using the conjugate-gradient algorithm with a force convergence threshold of 10^−3^ eV Å^−1^. We used the screened-exchange Heyd-Scuseria-Ernzerhof hybrid function (HSE06) to study the electronic properties of TlGaTe_2_. The screened parameter was set to 0.2 Å^−1^ and 30% of the screened Hartree-Fock (HF) exchange was mixed with the PBE exchange functional in the HSE06 method [12]. We applied a 10 × 10 × 10 Γ-centered Monkhorst-pack k-point mesh, which is used in HSE06 self-consistent calculations for TlGaTe_2_. For the calculation of vibrational properties, the software PHONOPY along with VASP, was utilized [13,14]. For vibrational properties, we used 2 × 2 × 2 supercell (64 atoms). Solving the Casida’s equation is the best approach for calculating the dielectric function [15]. We used a 16 × 16 × 16 -centered grid for both GW (green function with Coulomb interaction) and the Bethe-Salpeter equation (BSE). For all these calculations, a plane-wave cut off of 500 eV was used. The Raman activity was obtained by calculating the polarizability for the vibrational modes [16]. The IR spectra is simulated with the analytic formula of Baroni et al. (using the Born effective charge tensor) [17].

## 3. Results and Discussions

**Figure 1:**
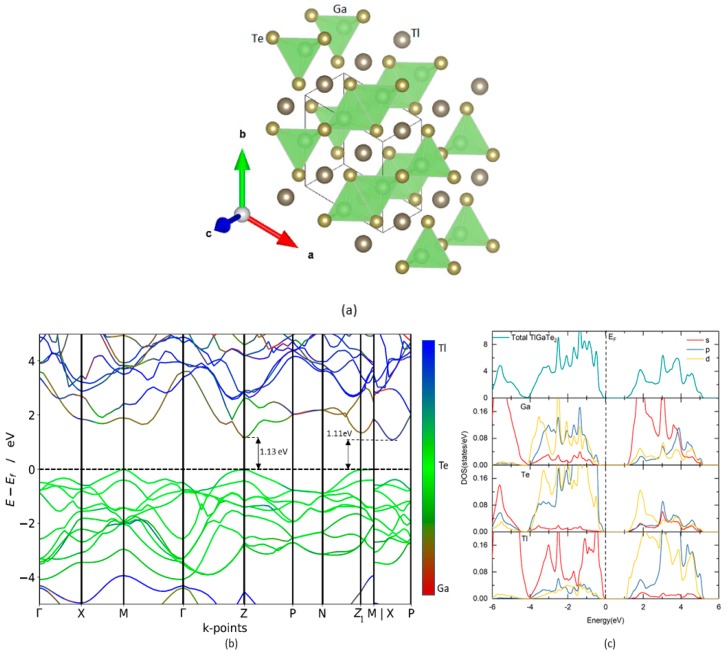
(**a**) Crystal structures of TlGaTe_2_. The illustration shows the legends for the different type of atoms; (**b**) calculated HSE06 electronic band structure of TlGaTe_2_; (**c**) total and site projected density of states of TlGaTe_2_. The Fermi level is set to zero. (colour code: red-Ga, green-Te, blue-Tl).

TlGaTe_2_ crystallizes in a tetragonal structure (I4/mcm, space group No. 140) as shown in Figure 1a. The calculated Ga-Te distance is 2.69 Å, and Tl-Tl bond length is smaller than the separation between Te and Tl atoms, which make up the GaTe_4_ tetrahedra (Figure 1a) [18]. The open-circuit voltage and short-circuit current of the solar cells are highly influenced by the band gap of the photoactive semiconductors. The detailed-balance limiting efficiency of an ideal solar cell is 32% for a material with the optimal band gap of 1.4 eV [19]. Band gap calculation using electronic band structure provides a promising opportunity to identify suitable PV materials. The band structure and site-projected density of states of dichalcogenide TlGaTe_2_ calculated with HSE06 are shown in Figure 1b,c, respectively. The valence band maximum (VBM) is located at the Z-point and Z1-point. The first conduction band minimum (CBM) is located along M-P directions and the second CBM is located at Z-point. In two different experimental studies, it was reported that the band gap energy of TlGaTe_2_ is 0.84 eV [20] and 1.2 eV [21]. The reason for this deviation is not known. Our calculations show that TlGaTe_2_ has an indirect band gap of 1.109 eV, which is higher than the mBJ calculation (0.94 eV) [22] but has a good agreement with an experimentally verified band gap value of 1.2 eV [21]. Also, we observe that TlGaTe_2_ exhibits a direct band gap of 1.129 eV at the Z-point and the difference between direct and indirect band gap is 20 meV. This phenomenon is verified further by a study on the optical properties of TlGaTe_2_ later in this article. The site-projected density of states shows that valence band maximum (VBM) is mainly due to Tl-s state with little contribution of Te-p state, while conduction band maximum (CBM) is mainly contributed by Ga-s state and Tl-d state in Figure 1c.

Since TlGaTe_2_ exhibits narrow indirect band gap at the Z1-point (Figure 1b), the VBM is flatter than the first CBM along M-P directions. Note that around Z-point, the VBM is somewhat flatter than the second CBM. Hence, it would appear that electrons are lighter than holes. The effective masses of holes and electrons have been calculated by the finite difference method [23]. The effective masses of TlGaTe_2_ calculated by HSE06 are listed in Table 1. For the indirect electronic transition, we found that the effective mass of electrons is 0.196 me (me–a mass of an electron) along the M|X-P direction and the effective mass of holes is 0.736 me along the Z1-M direction. It is interesting to note that Qasrawi et al. used the Hall effect measurement and reported the hole effective mass of TlGaTe_2_ to be 0.730 me [7], which is in good agreement with our calculated hole effective mass (0.736 me). For the direct transition, we noted that the EM of electrons is 0.378 me and EM of holes is 0.736 me at Z-point.

For comparison, the effective mass of electron such as silicon (Si) and gallium arsenide (GaAs) are 0.26 and 0.12 me [24], respectively. The effective mass (EM) of the electron is low for TlGaTe_2_ compared to Si. Thus, TlGaTe_2_ has high electron mobility than silicon, which would be useful for an efficient solar cells [25]. 

**Figure 2:**
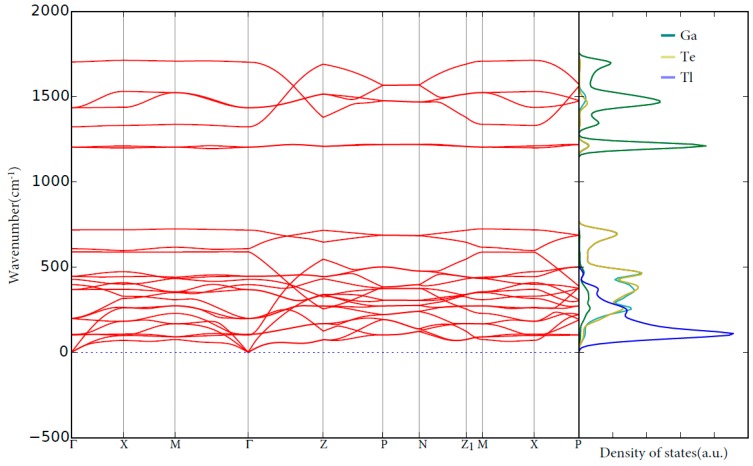
Calculated phonon spectra and site projected phonon density of states for TlGaTe_2_.

In order to understand the dynamical stability of the TlGaTe_2,_ we carried out the phonon calculations. The calculated vibrational spectrum curves together with the corresponding site projected density of states are plotted in Figure 2. The positive phonon frequencies suggest that TlGaTe_2_ is dynamically stable. The phonon frequencies in the TlGaTe_2_ have a separation between the low and high-frequency modes (the so-called phonon band gap). The vibrational modes are spread over from 0 to 1735 cm^−1^. As expected, based on the relationship between masses and frequencies given by equation [26], the heavier element thallium is normally connected with the lower frequencies. The lattice vibrational modes for Tl, Ga, and Te are present in the 0 to 450 cm^−1^ range, 1167 to 1735 cm^−1^ range and 0 to 767 cm^−1^ range, respectively.

To validate the mechanical stability of tetragonal-TlGaTe_2_, we calculated the single-crystal elastic constant tensor using the finite strain technique. This structure has six independent elastic constants namely C_11_ = 32.205, C_12_ = 15.894, C_13_ = 11.959, C_33_ = 58.188, C_44_ = 10.237_,_ and C_66_ = 12.009. The mechanical stability criteria for the tetragonal (type I) phase are given by [27]:

C_11_ − C_12_ > 0 (1)

2(C_11_ + C_12_) + C_33_ + 4C_13_ > 0 (2)

C_44_ > 0, C_66_ > 0 (3)

C_11_ + C_12_ − 2C_13_ > 0 (4)

All four conditions for the mechanical stability of tetragonal-TlGaTe_2_ given in Equations (1)–(4) are satisfied, and this finding indicates that tetragonal-TlGaTe_2_ phase is mechanical stable. From the calculated elastic constants, the bulk (B_v_, B_R_) and shear moduli (G_v_, G_R_) are calculated from Voigt–Reuss–Hill approximations [28,29]. Our calculated values of bulk moduli (B) and shear moduli are 22 GPa and 20.143 GPa from the elastic constants, respectively. The critical G/B value that differs the ductile and brittle materials is 0.5 [30]. As known, when the G/B value of materials is lesser than 0.5, then those materials are ductile—otherwise, they are brittle. The value of G/B for TlGaTe_2_ is 0.916, which shows that TlGaTe_2_ is brittle. 

**Figure 3:**
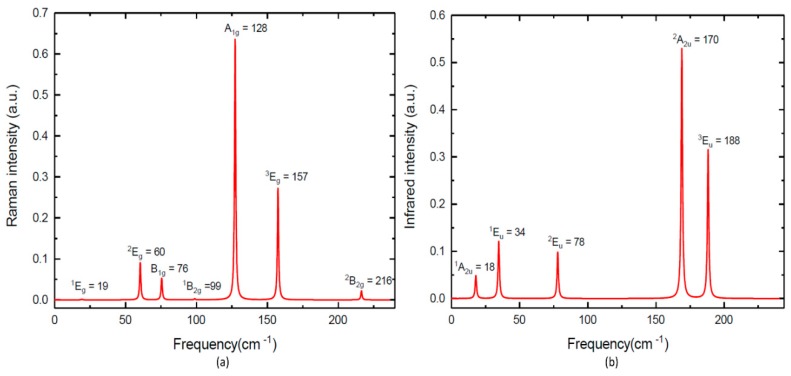
(**a**) Calculated Raman spectra of TlGaTe_2_; (**b**) calculated infrared spectra of TlGaTe_2_.

According to the crystal symmetry, we have the following modes and the selection rules for IR-active and R-active modes, Γ = A_1g_ (R) + 2A_2u_ (IR) + B_1g_ (R) + B_1u_ + 2B_2g_ (R) + 3E_u_ (IR) + 3E_g_ (R). As seen in Figure 3a and Table 2, we observed seven R-active modes in the Raman spectra for TlGaTe_2_, whereas Gasanly et al. [9] found only three out of seven predicated R-active modes. Our calculated R-active modes confirm the existence of three E_g_ mode with frequencies of ^1^E_g_= 19 cm^−1^ (16 cm^−1^ [31]), ^2^E_g_ = 60 cm^−1^ (67 cm^−1^ [9]) and ^3^E_g_ = 157 cm^−1^ (165 cm^−1^ [9]). Our calculated R-active modes confirm the existence of one A_1g_ mode with a frequency of A_1g_ = 128 cm^−1^ (135 cm^−1^ [9]) and three B_1g_ mode with frequencies of B_1g_ = 76 cm^−1^ (76 cm^−1^ [31]), B_1g_ = 99 cm^−1^ (99 cm^−1^ [31]) and ^2^B_2g_ = 216 cm^−1^ (210 cm^−1^ [31]).

From Figure 3b and Table 2, we observed five IR-active modes in infrared spectra calculation [17]. Our calculated IR-active modes confirm the existence of two A_2u_ mode with frequencies of ^1^A_2u_ = 18 (27 cm^−1^ [9]) and ^2^A_2u_ = 170 (175 cm^−1^ [9]), along with three E_u_ mode with frequencies of ^1^E_u_ = 34 (44 cm^−1^ [9]), ^2^E_u_ = 78(88 cm^−1^ [9]) and ^3^E_u_ = 188(192 cm^−1^ [9]). In Table 2, we presented our calculated values for R-active and IR-active modes along with results published earlier in [31] and the experimental values [9]. Note that our calculated R-active and IR-active modes are in better agreement with the experimental results obtained in [9] than [31]. However, the calculated phonon frequencies of TlGaTe_2_ are lower than the corresponding experimental phonon frequencies. The exchange correlation functional used in this study is not enough to estimate the transition pressure. On the other hand, it is very difficult to obtain a pure form of TlGaTe_2_ at ambient conditions due to experimental limitations and metastability.

**Figure 4:**
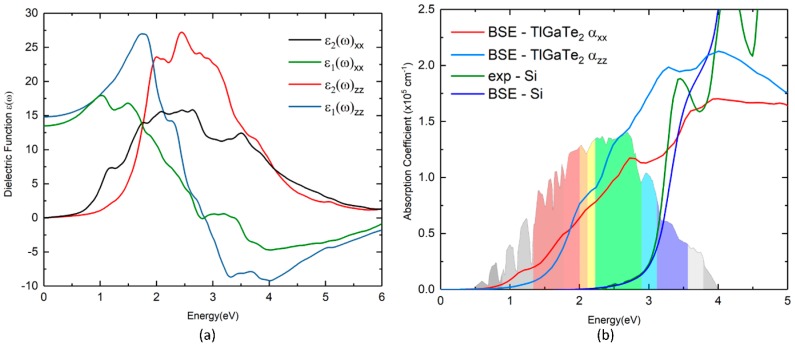
(**a**) Calculated dielectric function of TlGaTe_2_; (**b**) absorption coefficient of TlGaTe_2_.

The optical properties have major influence on the solar cell materials; thus, we study the absorption properties of TlGaTe_2_ material by employing optical dielectric function ε(ω) = ε_1_(ω) + iε_2_(ω). The dielectric function is contingent on the wavelength of the electromagnetic radiation, and it is related to the interaction between photons and electrons. The absorption coefficient of the material is highly influenced by the imaginary part ε_2_(ω), and it can be determined from the inter-band optical transitions by summing over the unoccupied states using the equation [32]. The real part ε_1_(ω) of dielectric function can be calculated from the ε_2_(ω) by Kramer-Kronig relationship [33]. The calculated imaginary part of the dielectric function and absorption coefficients of the TlGaTe_2_ compound are presented in Figure 4. According to the directional dependency of ε_1_(ω) and ε_2_(ω), we can conclude that TlGaTe_2_ is an anisotropic material to the optical properties. Therefore, we present both the real and imaginary parts of the dielectric function of TlGaTe_2_ in Figure 4 along x and z-directions. 

In Figure 4a, both the real and the imaginary part of the dielectric function are plotted against the photon energy. The absorption coefficients (α) for TlGaTe_2_ are calculated numerically with the inclusion of excitonic effects treated within the Bethe-Salpeter equation (BSE) method, which gives numerical results that are in better agreement with the experimental absorption spectra [34,35,36,37]. To achieve BSE calculation, we start from the DFT level, which permits one to obtain a ground state energy of the TlGaTe_2_. In the second stage, GW (G–Green function and W–Coulomb interactions) approximations are used to calculate the quasi-particle energies. The GW function fails to calculate the excitonic effect between electron-hole interactions. The electron-hole interactions are then included beyond GW approximations, and the BSE method is employed in order to get improved dielectric function for TlGaTe_2_. To shed light on the accuracy of the BSE method, we calculated the absorption coefficient for silicon and compared with the corresponding experimental results. In Figure 4b, both the numerical and experimental absorption coefficients for Si and TlGaTe_2_ are depicted in Figure 4b. Note that the calculated absorption coefficient based on the BSE method for silicon is almost similar to the experimental values obtained from [36].

From Figure 4a,b, it is clearly seen that TlGaTe_2_ exhibits absorption peaks at 1.13 eV along the x-direction, which has to be due to the direct band gap of 1.129 eV at Z-point (from electronic band structure). Figure 4b shows a high absorption coefficient in the visible region. Absorption peaks of TlGaTe_2_ are observed at 1.129, 2.02, 2.5 and 2.7 eV along both x and z-directions. When the photon energy is about 2.7 eV along the x-direction, and 2.5 eV along the z-direction, the absorption coefficient of TlGaTe_2_ reached a maximum in the visible region. The valence band maximum is mainly attributed to the states Tl-*s*, and thallium is a very good absorbing material in the visible region. Consequently, we observed that TlGaTe_2_ exhibits high absorption in the visible region. Due to the indirect band gap of silicon, the absorption occurs for the photon energies higher than 2.5 eV as shown in Figure 4b. The difference between the indirect and direct band gap in silicon is about 1.40 eV, which results in low absorption coefficients for silicon in the visible region. Our results show that TlGaTe_2_ has better absorption coefficients compared to silicon in the visible region.

## 4. Concluding Remarks

In summary, we can mention that the electronic band structure and optical properties of TlGaTe_2_ with accurate first-principle calculations were investigated. The calculated HSE06 electronic structures confirmed that TlGaTe_2_ exhibits an indirect band gap, with a value of 1.109 eV, at the Z_1_-point. However, TlGaTe_2_ also exhibits a direct band gap, with a value of 1.129 eV at the Z-point. The first absorption peak at a photon energy of 1.129 eV confirms this finding. Until now, numerical limitations in calculating the band gap energies in the range of a few meV difference have been leading to claims stating that the fundamental gap of TlGaTe_2_ is indirect. Our accurate calculations from employing BSE revealed that TlGaTe_2_ possesses an additional direct band gap of 1.129 eV with a difference of 20 meV compared to the indirect band gap. Our comparison of the calculated absorption coefficient of silicon and TlGaTe_2_ shows that TlGaTe_2_ has a better absorption coefficient than silicon in the visible region.

Our phonon calculations show that TlGaTe_2_ is dynamically stable as no imaginary frequency was observed. Our elastic constant calculations illustrate that TlGaTe_2_ is mechanically stable. Raman and IR calculations are carried out for TlGaTe_2_ and compared with previous experimental and calculated results. Our results seem to be in better agreement with the experimental results than any other calculated results published previously. Our detailed studies of electronic and optical properties of the ternary dichalcogenide material suggest that TlGaTe_2_ is a potential candidate for photovoltaic applications.

## Figures and Tables

**Table 1:** The calculated effective masses of TlGaTe_2_, where m*_hh_ and m*_e_ are the effective masses of heavy holes and electrons respectively. m_e_ is mass of the electron.

Compounds	Effective Masses	
	m*_hh_ m_e_	m*_e_ m_e_
TlGaTe_2_ (indirect electronic transition 1.109 eV)	0.736	0.196
TlGaTe_2_ (direct electronic transition 1.129 eV)	0.736	0.378

**Table 2:** Calculated phonon frequencies ω_cal_ (in this work and previous work [31]) and their experimental values ω_exp_ [9].

TlGaTe_2_-Phonon Activity	Phonon Frequencies (in cm^−1^)		
	ω_cal_ [31]	ω_cal_ (This Work)	ω_exp_ [9]
R-active ^1^E_g_	16	19	-
R-active ^2^E_g_	60	60	67
R-active ^3^E_g_	152	157	165
R-active B_1g_	76	76	-
R-active A_1g_	125	128	135
R-active ^1^B_2g_	99	99	-
R-active ^2^B_2g_	210	216	-
IR-active ^1^A_2u_	8	18	27
IR-active ^2^A_2u_	161	170	175
IR-active ^1^E_2u_	31	34	44
IR-active ^2^E_2u_	77	78	88
IR-active ^3^E_2u_	182	188	192
